# Resistant *C. albicans* implicated in recurrent vulvovaginal candidiasis (RVVC) among women in a tertiary healthcare facility in Kumasi, Ghana

**DOI:** 10.1186/s12905-024-03217-6

**Published:** 2024-07-19

**Authors:** Abena Kyeraa Sarpong, Hayford Odoi, Yaw Duah Boakye, Vivian Etsiapa Boamah, Christian Agyare

**Affiliations:** 1 Pharmaceutical Microbiology Section, Department of Pharmaceutics, Faculty of Pharmacy and Pharmaceutical Sciences, Kwame Nkrumah University of Science and Technology, Kumasi, Ghana; 2 Department of Pharmaceutical Microbiology, School of Pharmacy, University of Health and Allied Sciences, Ho, Ghana; 3 Pharmaceutical Microbiology Section, Department of Pharmaceutics, Faculty of Pharmacy and Pharmaceutical Sciences, Kwame Nkrumah University of Science and Technology, Kumasi, Ghana; 4 Laboratory Technology Department , Kumasi Technical University, Kumasi, Ghana

**Keywords:** Recurrent vulvovaginal candidiasis, Candida albicans, Antifungal susceptibility, Women’s health

## Abstract

**Background:**

Vulvovaginal candidiasis is a common fungal infection that affects the female lower genital tract. This study determined the major risk factors associated with vulvovaginal infection (VVI) in the Ashanti region of Ghana and also determined the antifungal resistance patterns of *Candida albicans* isolates to some antifungals.

**Methods:**

Three hundred and fifty (350) high vaginal swab (HVS) samples were collected from women who presented with signs and symptoms of VVI. A structured questionnaire was administered to one hundred and seventy-two (172) of the women. HVS samples were cultured on Sabouraud dextrose agar with 2% chloramphenicol. The polymerase chain reaction was employed to confirm *C. albicans*. Antifungal susceptibility testing was performed and the susceptibility of *C. albicans* isolates to fluconazole, clotrimazole, amphotericin B, nystatin, miconazole and 5-flurocytosine were assessed.

**Results:**

Vaginal infection was most prevalent amongst females in their reproductive age (21 to 30 years; 63.0%). The study found a significant association between vaginal infections and some risk factors such as sexual practices (*p* < 0.001), antibiotic misuse (*p* < 0.05), poor personal hygiene (*p* < 0.005) and birth control methods (*p* < 0.049). Out of the 350 HVS samples collected, 112 yielded yeast cells with 65 (58%) identified as *C. albicans*. The *C. albicans* isolates were resistant to 5’ flucytosine (100%), fluconazole (70%), voriconazole (69.2%), miconazole (58.5%) and nystatin (49.2%). *C. albicans* isolates were more susceptible to amphotericin B (53.8%) and clotrimazole (45.1%), although an appreciable number of isolates showed resistance (46.1% and 52.3%, respectively).

**Conclusion:**

There should be nationwide education on all associated risk factors of VVI. Also, use of the various antifungal agents in vaginal candidiasis should proceed after antifungal susceptibility testing to ensure efficacious use of these agents.

**Supplementary Information:**

The online version contains supplementary material available at 10.1186/s12905-024-03217-6.

## Background

Antifungal resistance is a developing problem and a major focus of the Antimicrobial Stewardship (AMS) efforts [1, 2]. *Candida* species cause the most common fungal diseases, with over twenty pathogenic species identified, among which *Candida albicans* is the most frequently isolated [3]. This species comprises 75% of the fungal species sampled from human specimens. *C*. *albicans* is one of 200 organisms belonging to the genus *Candida* [4]. In general, this organism lives as a non-threatening commensal entity in a variety of locations in the human host, most notably the oral cavity, epidermis, genitalia and gastro-intestinal tract [5]. Immunocompromised individuals are most often susceptible to *Candida* infections, which can lead to hepatosplenic abscesses, myocarditis, central nervous system or pulmonary infections during invasion [6].

Women who attend hospitals as a result of VVC are usually those who have suffered the infection and still suffer from it even after completing the therapy. This continuous recurring infection affects life of women especially with the associated signs and symptoms [7]. Disturbance and alteration of the vaginal microbiota contributes to vaginal infection [8]. Host related factors such as hormonal replacement therapy, pregnancy, immunosuppression and behavioural factors such as douching, use of contraceptives, sexual behaviour, tight clothing, poor hygiene and antibiotics usage have also been identified as contributing factors to recurrent vulvovaginal candidiasis [9].

There have been records of resistance of microorganisms to antimicrobial agents over the past years. These have resulted in a burden on both health care providers and patients including high cost of treatment and high mortality rates [10]. Various factors have been attributed to the occurrence of microbial resistance, including drug misuse and patients’ inability to complete antimicrobial therapy. It is currently believed that the increasing rate of recurrent VVC is partly due to the development of resistance against antifungal agents [11].

In Ghana, *C. albicans* has been reported to be the most prevalent *Candida* species to cause fungal infection [12, 13]. Whiles high susceptibility of *C. albicans* to fluconazole and voriconazole has been reported, resistance to other antifungal agents including amphotericin B, itraconazole and 5- flurocytosine have also been documented [12]. According to Feglo and Narkwa [12], voriconazole was the most active antifungal agent with no resistance whereas the overall resistance of the isolates to other antifungal agents ranged from 4.5 to 22.2%, which is a cause of concern. Another study in Ghana also reported amphotericin B as the most active antifungal agent against vaginal *Candida* isolates with susceptibility rate of 87.2%, although amphotericin B, fluconazole and itraconazole were the only antifungals used in the study [13]. Reports from Europe and Asia indicate high resistance rates of *C. albicans* and non-*Candida albicans* to the available antifungals including fluconazole and voriconazole [13].

Recurrent vulvovaginal candidiasis (RVVC) is a more serious clinical condition due to the recurrence of symptoms, with a strong negative impact on both work and social life [14]. *C. albicans* has not been identified to be resistant to fluconazole in previous studies in Ghana, although there is increasing trend of resistance in other countries. Reports from Europe and Asia indicate high resistance rates of *C. albicans* and non-*Candida albicans* to the available antifungals including fluconazole and voriconazole [15, 16], whereas *C. albicans* has not been found to be resistant to fluconazole in previous studies in Ghana.

## Methods

### Study site and study design

A cross-sectional study was conducted on 350 women with episodes of recurrent vulvovaginal infection (RVVI) between the ages of 12 to 80 years. They had reported to the Obstetrics & Gynaecology unit at the Komfo Anokye Teaching Hospital (KATH) from February 2017 to March 2018. KATH is a 1200 bed-capacity healthcare facility and a major referral centre serving the southern and middle belts of Ghana. The Obstetrics and Gynaecology Department at KATH specializes in areas such as Uro-gynaecology, Gynae-oncology, Reproductive Health and Family Planning, Feto-maternal Medicine and Endocrinology and Infertility. They attract a lot of health conditions related to reproductive health in women making the centre ideal for the conduct of the studies (The Ministry of Health and Ghana Health Service, 2017; Komfo Anokye Teaching Hospital, 2013).

### Study population, participant’s selection and recruitment

The study population included women with signs and symptoms of RVVI, who visited any of the specialized units of the Obstetrics and Gynaecology Department of KATH. Participants who gave consent were enrolled on the study. For minors, consent was obtained both from the participant and the parent or guardian before enrolment in the study. Women with vaginal related complication such as vaginal bleeding were excluded from the study. Those on antifungal or antibiotic therapy were also excluded from the study.

### Specimen collection and study procedure

Two high vaginal swab (HVS) specimens were obtained through a purposeful sampling of 350 women aged between 12 and 80 years. The HVS samples were taken by trained technicians with the aid of a speculum and a sterile cotton tipped swab in an enclosed room. Samples collected were immediately transferred into sterile swab containers and sealed. Respective swab of each participant was labelled with a unique pathological code and immediately processed for laboratory analysis. Among the 350 women who were recruited for the study, 172 of them gave complete information on participants’ socio-demographics, level of immunity, antibiotic use, sexual practices, personal hygiene, toilet facility usage, douching practices and previous history of genital tract infection through face-to-face interviews using a structured questionnaire (see Supplementary File 1). Some participants either refused to respond to questions from the questionnaires or did not complete the questionnaires, all participants allowed their samples to be taken for culture, isolation and susceptibility testing.

### Isolation and maintenance of yeast

**Fig. 1:**
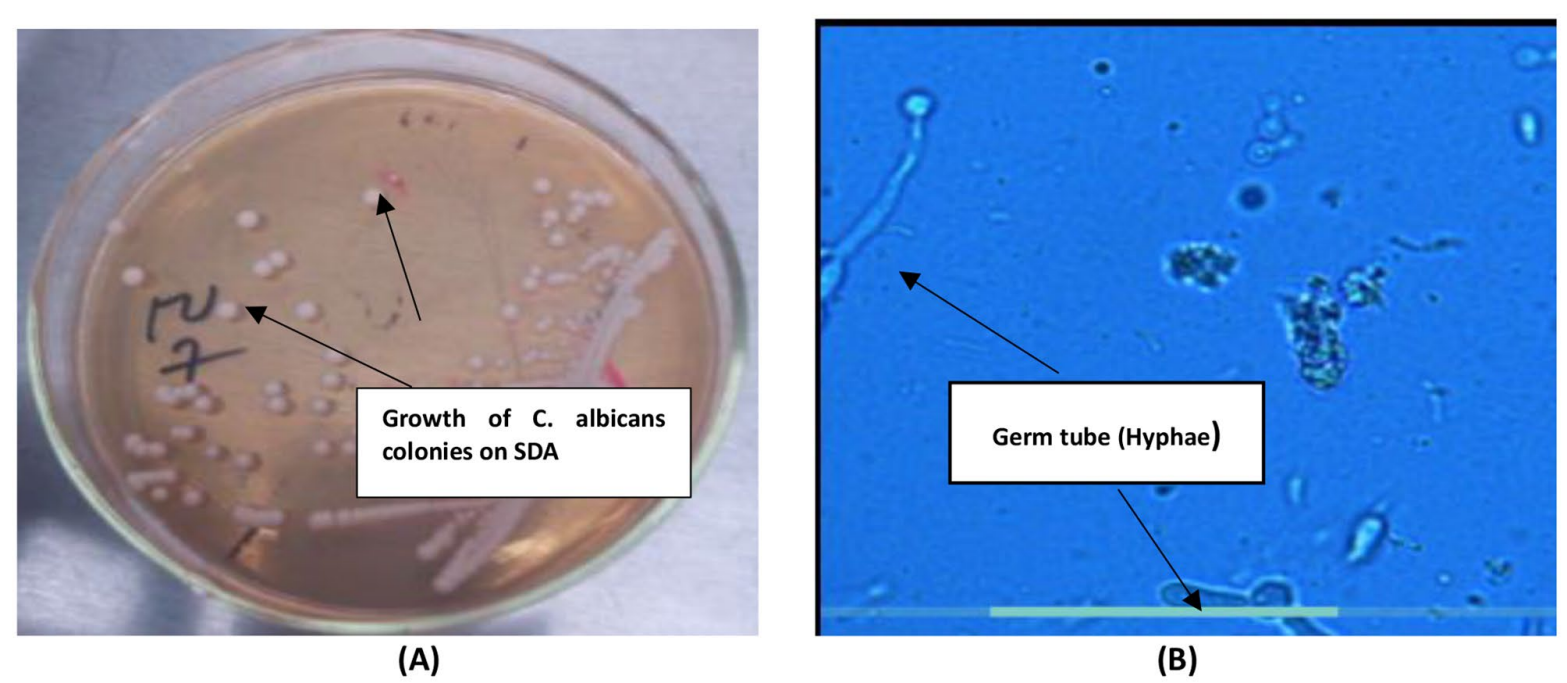
**A**: Photograph of *Candida species* (pale cream, smooth, glistening, convex colonies) on Sabouraud Dextrose Agar supplemented with 2% chloramphenicol after 48 h of incubation **B**: Micrograph of Germ tube formation in *Candida* species; *C. albicans* (short, slender tube structure without constrictions- Germ tube positive)

HVS samples were processed at the Microbiology laboratory of the Komfo Anokye Teaching Hospital within 1 h of collection. The swabbed sample were used for culture and direct Gram smear for Gram-positive oval-shaped organisms with buds and/or pseudohyphae/hyphae (Fig. 1B). Individual sterile cotton swabs were aseptically inserted into 12 mL sterile nutrient broth in a test tube under a Skan biosafety cabinet. It was quickly stirred in the broth and gently removed by rotating on the walls of the test tube above the level of the volume of broth. The inoculated broth were then incubated at 37 °C for 24 h for growth of organisms. After incubation, 10 µl of culture in broth was aseptically streaked on 20 mL Sabouraud dextrose agar containing 2% w/v chloramphenicol (TPC, India). The inoculated agar plates were then incubated aerobically at 37 °C for 48 h. All isolates were transferred into 20% w/v glycerol broth and stored at -80 °C until needed [17]. Colonies on SDA plates were subjected to colony identification (Fig. 1A) and gram staining for yeast-like cells. *C. albicans* yeast-like cells were identified using the germ tube test (Fig. 1B) and polymerase chain reaction.

### Germ tube formation assay and PCR confirmation of *C. albicans*

Germ tube formation (Fig. 1B) is a unique characteristic of *C. albicans* and *C. dubliniensis* amongst the other *Candida* species. Yeast cells of *C. albicans* are able to form hyphae with spores with or without constriction [18]. Stored isolates were revived by inoculating on sterile Sabouraud dextrose agar (Oxoid, R01768) containing 2% w/v chloramphenicol which was incubated at 37 °C for 48 h. A small discrete pure colony of yeast was transferred into sterile tubes containing 0.5 mL human sera. The mixture was incubated at 37 °C for 2 to 3 h. A drop of the resulting suspension was examined microscopically at a magnification of x10 and x40. The appearance of small or long tube-like filaments projecting from the cell surface confirmed formation of germ tubes which is characteristic of *C. albicans* [19]. The procedure was carried out in triplicate for all isolates.

**Fig. 2:**
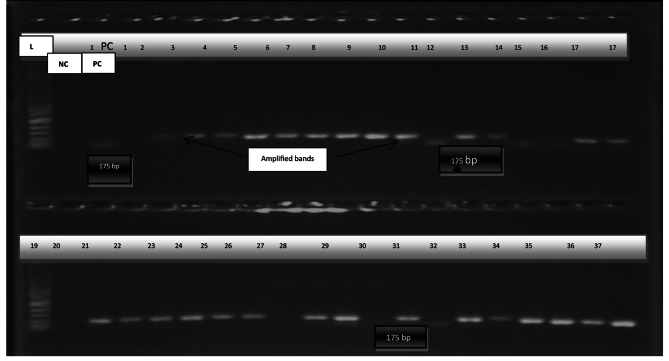
Electrophoretic gel image showing the 175 bp PCR amplicon of the rRNA gene of *C. albicans*. L-100 bp DNA ladder, NC: Negative control, PC: Positive control

The presumptive isolates were confirmed by the amplification of *C. albicans* 25S rRNA gene to produce a 175-bp DNA fragment. DNA was extracted using the modified cetyltrimethylammonium bromide (CTAB) method as described by [20]. The PCR amplifications were performed in a 15 µL reaction mix which consisted of 10 ng of genomic DNA, 3 µL of 5x PCR buffer (mi- red load Taq mix, Metabion Int), 0.2 µL each of the forward primers, CALBF (5’-TGTTGCTCTCTCGGGGGCGGCCG-3’) and reverse primers CALBR: 5’- AGATCATTATGCCAACATCCTAGGTTAAA − 3’ (Inqaba Biotec, South Africa). The suspension was adjusted with PCR grade water to make up the final volume. For polymerase chain reaction (PCR) amplification, the DNA template was initially denatured at 94 °C for 30 s, followed by 35 cycles of denaturation at 94 °C for 60 s, annealing at 55 °C for 30 s and extension at 72 °C–1 min. Finally, the products were extended at 68 °C for 5 min. The PCR products were then analysed using agarose gel electrophoresis in 1x Tris acetate acid EDTA (TAE) buffer on a 2% w/v agarose gel at 100 V and visualized using a transilluminator (Fotodyne, 60-2105) (Fig. 2).

### Antifungal susceptibility testing of *C. albicans* isolates

**Fig. 3:**
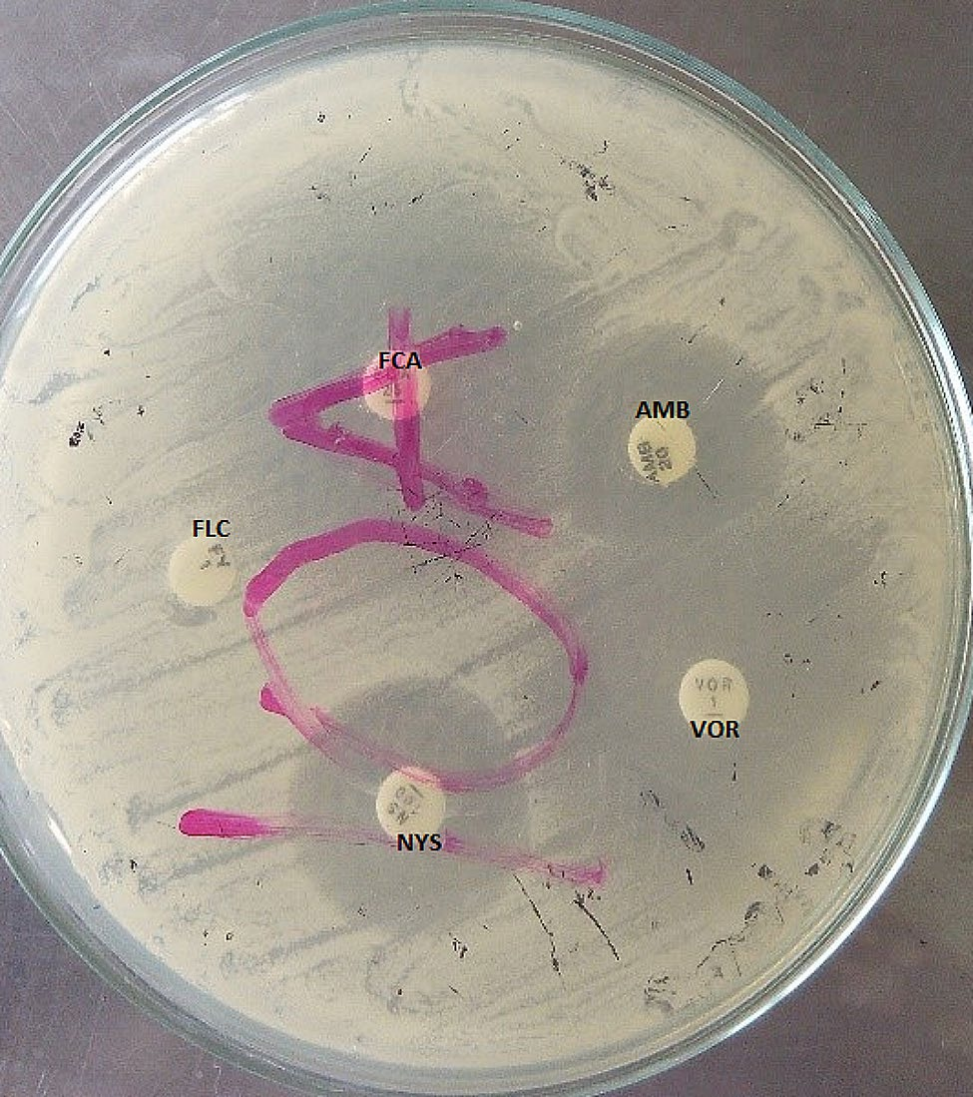
Photograph of antifungal susceptibility testing of amphotericin B (AMB; 20 µg), fluconazole (FCA; 25 µg), nystatin (NYS; 100 units), voriconazole (VOR; 1 µg) and 5-flurocytosine (FLC; 1 µg) showing zone of inhibition on Muller Hinton agar after 18 h of aerobic incubation at 37 °C

Susceptibility of *C. albicans* isolates to the selected antifungals was determined by the Kirby-Bauer disk diffusion testing according to approved methods of the Clinical Laboratory Standards Institute [21]. The antifungals used for the susceptibility of the *Candida* isolates were amphotericin B (AMB; 20 µg), fluconazole (FCA; 25 µg), nystatin (NYS; 100 units), miconazole (MCL; 10 µg), clotrimazole (CTM; 10 µg), voriconazole (VOR; 1 µg) and 5’ flucytosine (FLC; 1 µg) (Oxoid Ltd, Basingstoke, UK) [21, 22]. The International Unit of Nystatin is defined as the activity in 0.000333 mg of the International Standard nystatin [23]. An inoculum size for susceptibility test was standardized to 0.5 McFarland in a calibrated densitometer (Grant bio-DEN-1). Within 10 min after adjusting the turbidity of the inoculum suspension, a sterile cotton tipped swab (Fisherbrand™, FIS#22-363-173) was dipped in the solution, rotated several times and pressed firmly on the inside wall of the tube above the liquid. The swabs were streaked over the surface of a dried 20 mL Mueller-Hinton agar plate (Oxoid, CM0337). Streaking was rotated three times to ensure even distribution of the inoculum. The standard antifungal discs were then applied on the surface of the inoculated agar plate using a 90 mm 6-place disc dispenser (Oxoid, ST8090); and incubated at 37 °C for 18 h (Fig. 3). The zones of inhibition for each of the antifungal agents were measured in millimetres using a measuring ruler and compared with breakpoint values according to reference values from CLSI [20].

### Data collection, management and analysis

The clinical and laboratory data were entered into an MS-office Excel software Version 1908 (Microsoft Corporation Copyright 2016). Data was later analysed using Statistical Package for the Social Sciences Version 22 (SPSS, SPSS Inc., Chicago, IL, USA). Susceptibility data were compared by using Chi-square analysis with GraphPad Prism version 5.0 (Graph Pad Software, San Diego, CA, USA). A level of significance (*P*-value) < 0.05 were considered statistically significant.

## Results

### Participant characteristics and patient associated risk factors for VVC

Demographically, participants with signs and symptoms of vaginal infection were mainly between the ages of 21 to 30 years (63.0%), followed by those between the ages of 31 to 45 (24.9%). Participants between the ages of 46 to 60 years (1.7%) and 61 to 80 years (1.7%) were observed to have a lower prevalence of vaginal infection (Table 1). Most of the respondents had experienced tertiary (48.3%) and secondary school (38%) education (Table 1). Most of the participants involved in this study were also single (56.4%) (Table 1).

**Table 1:** Demographic characteristics and respondent health status

Description		Frequency (*n*)	Percent (%)
Age category and prevalence of infection			
	12–20	15	8.7
	21–30	109	63.4
	31–45	43	25.0
	46–60	3	1.7
	61–80	2	1.2
(*p* > 0.05)			
Level of education of respondents			
	JHS	19	11.0
	SHS	66	38.4
	O/A level	4	2.3
	Tertiary	83	48.3
Marital status of respondents			
	Married	73	42.4
	Single	97	56.4
	Divorced	2	1.2
Diagnosed of chronic disease			
	Yes	15	8.7
	No	157	91.3
Type of chronic disease			
	Diabetes	6	40.0
	HIV	0	0.0
	Hypertension	9	60.0
Pregnancy status of participants			
	Yes	14	8.1
	No	158	91.9
Hormonal disorder of participants			
	Yes	38	22.1
	No	134	77.9
Birth control methods of respondents			
	Oral contraception	117	68.0
	Diaphragm	9	5.2
	Spermicide	12	7.0
	None	21	12.2
	Other	13	7.6
Association between Age Category and Current Sexual Activity			
	**Yes**	**No**	**Total**
12–20	9	6	15
21–30	94	14	108
31–45	37	6	43
46–60	3	0	3
61–80	3	0	3

### Association between frequency of vaginal infection and related causes

#### Chronic diseases

There was a low prevalence (8.7%) of chronic diseases among participants with vaginal infection. Diabetes (40%) and hypertension (60%) were the main diseases reported by participants (Table 1). There was a low prevalence of chronic diseases among the participants who reported with vaginal infections (8.7%).

#### Type of toilet facility

In this study, there was no significant association between the type of toilet facility used and the frequency of vaginal infection (Table 2). Sixty-five (37.7%) of the respondents used private water closet as their toilet facility, followed by the public pit latrine (36.6%).

#### Antibiotic use

Antibiotic use by participants had a significant association (*p* = 0.001) with vaginal infection. Some of the participants (24.4%) had vaginal infections with non-frequent use of antibiotics while others (53.4%) had experienced recurrent infections in the last 12 months with occasional use of antibiotics. Participants who frequently used antibiotics (15.7%) experienced most recurrent vaginal infection (14.5%).

#### Underwear wash after removal

The period in which participants wash their underwear had a significant association (*p* = 0.005) to the frequency of vaginal infection (Table 2). The study revealed that ninety-nine (57.5%) participants who washed their underwear 2 to 3 days after removal were more likely to develop vaginal infections once or 2 to 3 times in a year. For participants who washed their underwear a day after removal, vaginal infections were experienced by 17.4% with 6.3% of them reporting to have experienced more than one vaginal infection in the past 12 months. Vaginal infection was experienced in participants who washed their underwear immediately after removal (9.8%) with one participant experiencing recurrent infections in the past 12 months.

#### Mode of drying underwear

The mode of drying underwear contributed significantly (*p* = 0.005) to the occurrence of vaginal infection. One hundred and twenty-nine (129, 75%) respondents dried their underwear outdoors in the sun yet 31% of them had experienced recurring infection. Thirty-six (36, 20.93%) of the respondents who dried their underwear outdoors but not in the sun also had vaginal infection occurring over the last 12 months with only 1.7% experiencing a recurrent infection. Six (6) of the respondents who dry their underwear indoors had only one of them experiencing recurrent infection (Table 1).

#### Sexual practices

There was a significant correlation between the number of sexual partners, frequency of sexual intercourse (*p =* 0.001) and the frequency of vaginal infection. Females with only one sexual partner or more than one sexual partner were at risk of contracting vaginal infection. Of the 172 respondents, 107 (62.2%) had one sexual partner with 16% of them reporting to have had recurrent infections in the past 12 months. Fifty-three (53, 30.8%) respondents had more than one sexual partner, with 27 (15.6%) experiencing recurrent infections in the last 12 months. Six (6) respondents had never had a sexual partner but had suffered recurrent infection in the last 12 months. Majority of the females (62%) had sexual intercourse once a week with 13% of them having a recurrent infection. Thirty (30, 18%) of these respondents have sex 3–6 times weekly and 10.4% reported of recurrent infections in the last 12 months. On the other hand, some (17%) of the respondents reported to have had no sex in the last 3 months, nonetheless 5% of them had recurring vaginal infections.

#### Douching practices

There was a significant (*p =* 0.001) relationship between vaginal cleaning methods and vaginal infection the frequency of vaginal infection. Eighty-nine (89, 52%) of the respondents washed their vagina with water only and 32.7% of them experienced recurrent infection. Seventy-eight (78, 45%) of the females washed their vagina with soap and water but only one reported to have experienced a recurrent infection in the last 12 months. Four (4) of the females used vaginal cream and antiseptics in washing the vagina (4, 2.3%).

Other Host Related Factors: Hormonal changes occur during pregnancy and immunity level is generally considered low during the first trimester of pregnancy (Vroumsia et al., 2013). Increase hormonal levels such as oestrogen, reduces the ability of vaginal epithelial cells to inhibit yeast cells thereby increasing the adhesion of Candida cells, this allows the formation of hyphae resulting in the reduction of vaginal immune response. Fourteen (14) participants representing 8.1% of the total participants reported that they were pregnant (Table 1). Additionally, thirty-eight (38, 22.1%) of the participants reported hormonal disorders (*p* = 0.002) (Table 1), while majority of the participants reported that they employed oral contraceptives (68%), spermicides (7%), diaphragms (5.2%) and other methods (7.6%) of contraception (Table 1). Such birth control methods may serve as a reservoir for the adhesion of yeast to form biofilms. Twenty-one (12.2%) participants did not employ any birth control method.

**Table 2:** Association between frequency of vaginal infection and related causes

Description	Frequency of Vaginal Infection in the Last 12 months									*p*-value
	*N*	Never		Once		2–3 times		≥ 4 times		
**Type of toilet facility frequently used and frequency of** **Vaginal Infection in the Last 12 months**										0.1
Public water closet	35		1		21		13		0	
Public pit latrine	63		3		43		13		4	
Private but shared water closet	65		7		32		23		3	
Private but shared pit latrine	9		0		7		2		0	
**Antibiotic use and frequency of vaginal infection**										0.001
Never	43		4		21		15		3	
Never	43		4		21		15		3	
Sometimes	102		5		59		36		2	
Frequently	27		1		1		18		7	
**Underwear Wash practices after Removal and frequency of** **Vaginal Infection in the Last 12 months**										0.005
Immediately after removal	17		1		15		1		0	
A day after removal	49		8		30		7		4	
Two to three days after removal	99		1		55		40		3	
A week or more after removal	7		1		3		3		0	
**Mode of Drying Underwear and frequency of** **Vaginal Infection in the Last 12 months**										0.005
Indoors	7		0		6		0		1	
outdoors not in the sun	36		2		31		2		1	
outdoors in the sun	129		9		66		49		5	
**Weekly Frequency of Sexual Intercourse during the** **Last 3 Months and frequency of Vaginal Infection in the Last 12 months**										0.005
Never	17		3		9		4		1–29	
Once a week	103		6		72		23		2-2.4	
3 to 6 times a week	30		1		12		14		3–56	
More than 7 times a week	2		0		1		1		0	
Others	14		1		3		9		1	
**Number of Sexual Partners and frequency of** **Vaginal Infection in the Last 12 months**										
1	107		7		71		28		1	
2–3	42		4		16		18		4	
4–6	10		0		8		0		2	
> 6	1		0		0		1		0	
None	6		0		2		4		0	
**Douching Practices and vaginal infection in the last 12 months**										
Only water	89		3		31		49		6	
Water and soap	78		8		69		1		0	
Antiseptics	3		0		2		1		0	
Feminine wash and vaginal creams	1		0		0		0		1	

N = total respondents

### Prevalence of *C. albicans* in HVS samples

Three hundred and fifty (350) clinical samples were cultured on Sabouraud dextrose agar. One hundred and twelve (112) yeast cell isolates were observed under the microscope of which 74 isolates appeared as germ tubes characterized by hyphae production. Growth of *C. albicans* on Sabouraud dextrose agar produced whitish or creamish colonies. A presumptive identification was performed by observing colony colour and morphology and yeast cell observation using microscopy. The microscopic formation of germ tube was characterized by spore formation without septum and constriction at the junction between the cells, with elongated hyphae. Germ-tube was indicative of *C. albicans* or *C. dubliniensis*. Sixty-five of these isolates were confirmed as *C. albicans* through PCR (Fig. 2).

### Susceptibility of *C. albicans* Isolates to antifungals

**Fig. 4:**
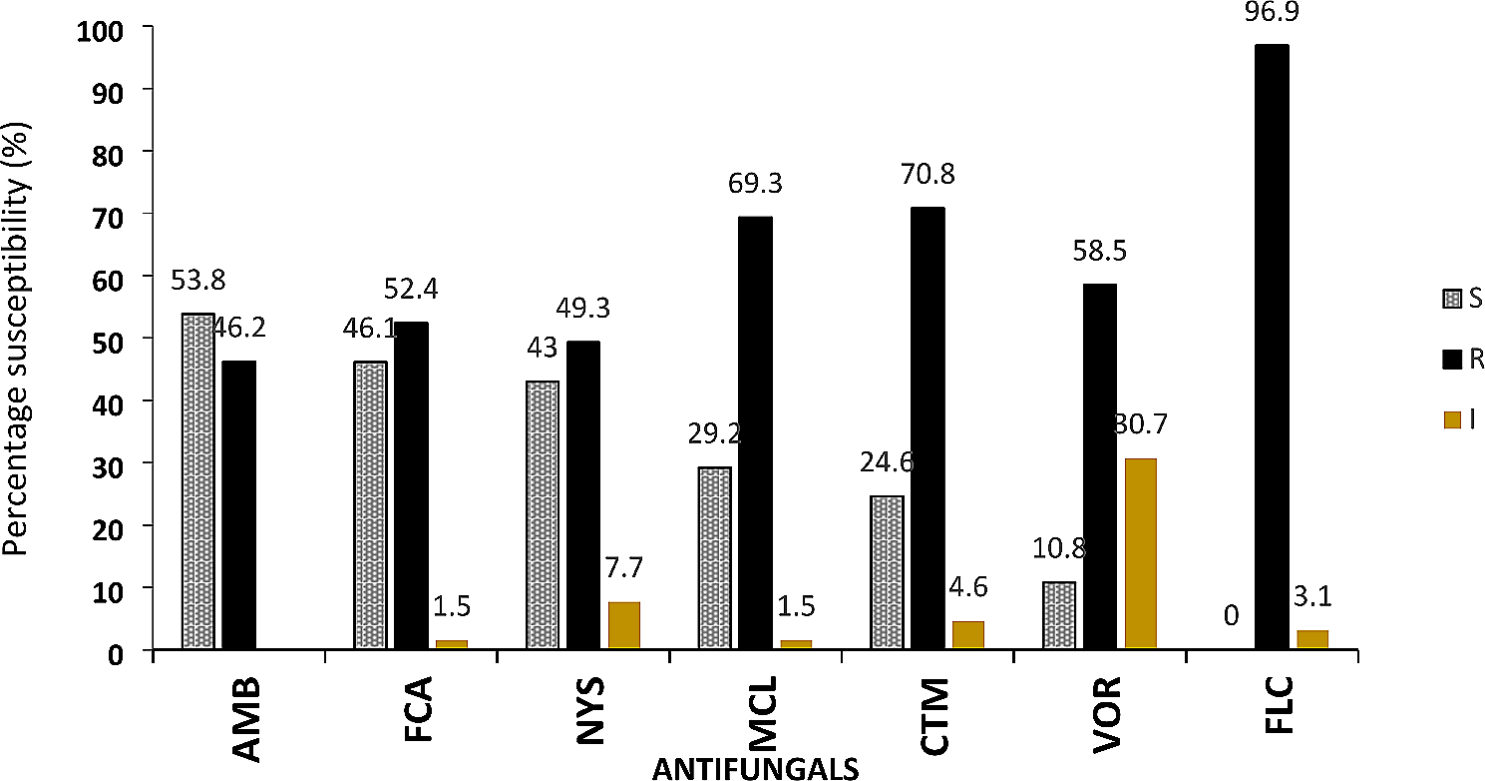
Susceptibility of antifungal agents against***C. albicans.*** AMB: amphotericin B 20 µg; FCA: fluconazole 25 µg; NYS: nystatin 100 units; VOR: voriconazole 1 µg and FLC: 5-flurocytosine 1 µg

The susceptibility rate of the *C. albicans* isolates to the selected antifungals ranged from 10.7–53.8% while the resistance rate of the isolates ranged from 46.1–96% (Fig. 4). The *C. albicans* isolates were most susceptible to amphotericin B (53.8%) (Fig. 4). The isolates were most resistant to 5’ flucytosine (96.9%) but resistance to fluconazole (70%), voriconazole (69.2%), miconazole (58.4%), nystatin (49.2%) and clotrimazole were also relatively high (Figs. 3 and 4).

## Discussion

Vulvovaginal candidiasis affects women all over the world. It usually occurs amongst women in their reproductive ages and is mostly caused by the fungal species *Candida albicans* [20]. The infection often lead to genital discomfort, reduced sexual pleasure and activity and psychological distress [24]. Unfortunately, there are limited reports on risk factors contributing to VVC and antifungal resistance in Ghana.

Majority of the participants in our study fell within the reproductive age ranges of 21 to 35 years (63%), and 36 to 45 years (24.9%), consistent with findings reporting VVC occurrence in 60 to 75% of females in these reproductive age groups [25, 26]. Furthermore, sexually active women are more prone to vaginal infection [27, 28]. Pathogens that cause these vaginal infections are polymicrobial and may cause a change in the vaginal microbiome with a massive increase of facultative and obligate anaerobic bacteria (mainly *Gardnerella spp*.) and a loss of lactobacilli [29]. The two categories of risk factors that contribute to the development of vaginal infection are host related risk factors and behavioural risk factors [30, 31]. Host related factors include pregnancy, hormone replacement, uncontrolled diabetes mellitus, birth control methods, immunosuppression and genetic predispositions while behavioural risk factors include poor personal hygiene, frequent antibiotic use, sexual practice and douching [24, 31].

Pregnant women and individuals with weak immune system and chronic conditions have been identified to be at high risk of contracting VVC. 8% (8%) of the participants were pregnant whereas 8.7% had an underlying disease. Vromsia et al. [32] reported a high prevalence of VVC amongst pregnant women in Cameroon whereas Disha and Haque [26] also reported diabetes as the most common risk factor of VVC followed by pregnancy among some females in a tertiary care hospital. The number of pregnant women and those with underlying disease condition such as diabetes in this study was relatively lower as compared to that reported by Disha and Haque [26]. This could be as a result of the special clinics such women visit regularly and as a result are always monitored by their clinicians. This discrepancy could also be as a result of differences in sample sizes employed in the two studies.

Some participants (22.1%) had conditions such as hormonal disorder and 87.8% were on a birth control method. According to Bruna et al. [31] and Odaranle et al. [24] some birth control methods such as diaphragm, oral contraceptives and intrauterine device increases *Candida* adhesion and hyphae formation and decrease vaginal immune response. They also act as reservoir where yeast adhere and form biofilms.

There was no significant association between the frequency of vaginal infection and the kind of toilet facility participants used. Those who used private but shared water closet, public water closet, public pit latrines, or private but shared pit latrines represented by 37.7%, 20.3%, 36.6% and 4.0% respectively, were all at risk of being infected. This is consistent with the report from Nsofor, Obijuru & Ohalete (2016) [32] which indicated that the frequency of getting *Candida* infection was not significantly different between squat and sitting toilets.

Participants who had one sexual partner and those who had multiple sexual partners were also at risk of getting vaginal infection. According to Bruna et al. [31] yeast cells are able to metabolize spermicidal compounds which allows an increase in adhesion. A high frequent sexual intercourse normally disturbs the vaginal microbiota. There was also a significant association (*p* = 0.001) between the number of sexual partner and vaginal infection. This result is consistent with reports by Bertini [33] and Bruna et al. [31] which indicates that sexual behaviour is a major risk factor for vulvovaginal infection.

Antibiotic use has been one of the major factors associated with *Candida* infection, since antibiotics kill the normal flora including *Lactobacillus* that protects the vagina environment from the overgrowth of unwanted organisms. In this study, antibiotic use had a significant (*p* = 0.001) association with vaginal infection. According to Chen et al. [34] the presence of a bacterial infection in the vaginal area is likely to predispose women to VVC after treatment of the bacterial infection with antibiotics. VVC is a common side effect of bacterial vaginosis treatment, especially with metronidazole or clindamycin, and women who frequently take these antibiotics are likely to develop VVC [34]. Even though sale of antimicrobials in Ghana is regulated, it is still easier for individuals to purchase prescription drugs over the counter. Hence, most females self-medicate before visiting the hospital which may affect the normal flora in the vagina and predispose participants to overgrowth of *C. albicans* leading to vaginosis.

A higher number of *Candida* spores in the vaginal environment facilitates the invasion of the organism leading to VVC, hence, personal hygiene plays a crucial role in preventing vaginal infections [16]. Female personal hygiene practices including bathing twice a day, shaving the pubic area frequently and wearing clean underwear each day are necessary for the prevention of vaginal infections [35]. The present study revealed that participants who washed their underwear a day or more after removal (57.5%) were more exposed to vaginal infections (*p* = 0.005), consistent with the findings of Hamed [35]. These findings strongly suggest that, underwear must be washed immediately after removal as a preventive measure to vaginal infections.

Some studies have reported a significant relationship between the mode of drying underwear and a reduction in the occurrence of vaginal infections [11, 26, 27]. Moist environments are known to promote microbial growth, particularly fungi, as moisture facilitates spore survival and proliferation. As these spores survive, the fungi begin to spread, creating more spores which spread quickly on surfaces [36]. In the present study, however, participants who dried their underwear outdoors in the sun and those who dried their underwear indoors were both at risk of getting vaginal infections (*p* = 0.005).

Sexual practice has been considered a major risk factor associated with vaginal infection. Frequent coitus disturbs the ecosystem of the vagina. From the study, sexual practices had significant correlation with vaginal infection (*p* = 0.001). In all, 56% of participants who engaged in sexual intercourse 3–6 times a week had experienced 2 to 4 vaginal infections within 12 months. A study conducted by Bruna et al. (2015) indicates that a change in the pH of the vaginal environment as a result of frequent sexual activity could cause the death of some normal vaginal flora such as *Lactobacillus* thereby allowing for the overgrowth of pathogenic organisms such as *C. albicans* [37].

Douching is the process of washing the inside and outside environment of the vagina with water or chemicals. Douching over the years has been associated with vaginal infection [32]. Chemicals such as vaginal washes, soaps and antiseptics used in douching could alter the vaginal pH and destroys the normal vaginal flora such as *Lactobacillus*, which protects the vaginal environment. In this study, there was a significant correlation between douching and vaginal infection (*p* = 0.001). The results of this study is consistent with that reported by Vroumsia et al. [32] which indicated that douching is a prominent risk factor for vulvovaginal candidiasis. Washing the vagina with only water is recommended since that will have no alteration in the vaginal environment [35].

The prevalence of *C. albicans* causing vaginal infection was high compared with non-*Candida albicans.* Of the 350 high vaginal swab samples collected, 65 (18.5%) isolates were confirmed as *C. albicans.* In a study carried out on women visiting a gynaecological clinic in Kumasi, Ghana, *C. albicans* was reported to be the most predominant *Candida* species (21%) causing vaginal candidiasis [12, 13]. This is consistent with the findings of this study since *C. albicans* was the most common (58%) organism amongst the yeast cells isolated. The above consistency notwithstanding, factors unaccounted for in this study; including nutritional, HIV and cancer chemotherapy management status are known to predispose individuals to fungal infections [6].

According to Abruquah [13], susceptibility of the isolates to antifungal agents ranged from 66.7 to 87.2% for amphotericin B, fluconazole and itraconazole. *C. albicans* was reported to be more susceptible to amphotericin B with sensitivity of 87.2%. This observation is in line with results of the present study as amphotericin B was more active against the isolated species (53.8%), though there was an appreciable decline in sensitivity of the isolates to amphotericin B which could be due to the evolution of resistance genes in *Candida albicans* strains. Resistance to the polyene antifungals, amphotericin B and nystatin involve alteration of the sterol synthesis pathway, thereby limiting the amount of sterols present in the cell membrane [38].

The findings from this study are also consistent with that of Siakwa et al. [39] with amphotericin B and clotrimazole showing susceptibility results of 53.8% and 45.1%, respectively. Though clotrimazole is known as one of the standard antifungal drugs used in the treatment of vulvovaginal infection, it has a fungistatic effect on fungal cells. Related reports from other African countries have indicated high prevalence of *Candida albicans* (71.7%) and non-albicans (24.1%) infections resistant to antifungals such as fluconazole [40] whereas other *Candida spp*. isolated in hospitals in Ghana have also been found to be resistant to antifungals such as voriconazole, fluconazole and nystatin [41].

Resistance for azoles may be due to changes in the 14-α-demethylase gene, changes in the pathway of sterol synthesis, reduction or overexpression of the target enzyme, and increased activity and number of drug efflux pumps [42, 43]. There was a high rate of resistance of *C. albicans* to the antifungal drug 5-Flurocytosine (96.9%), which could be as a result of the bacteriostatic effect of the agent to *C. albicans*. Deficiency in the enzymes necessary for cellular transport, uptake and metabolism. It could also be as a result of increased synthesis of pyrimidines, which compete with 5-Flurocytosine during RNA synthesis, diminishing its ability to affect RNA synthesis [43, 44].

### Strengths and limitations

The strength of this study lies in the establishment of the prevalence of recurrent vulvovaginal candidiasis among several women from a wide age range (12 to 80 years) along with presenting factors that could influence recurrence, including personal hygiene factors and sexual practices habits such as number of sexual partners and frequency of sexual intercourse. Some limitations, however need to be acknowledged. First, the survey relied solely on patient questionnaires. Also, only *Candida albicans* species were identified from the HVS. Other pathogens (either by themselves or in biofilm consortia) could be involved in vaginal infections; thus, it is possible that the prevalence of RVVC may have been underestimated due to such missed cases. Additionally, there is no linkage between patient details and the antifungal susceptibility results. Further studies to consider the above are thus warranted.

## Conclusion

Vaginal infection is most prevalent amongst females in their reproductive age of 21–30 years. There is a significant association between behavioural related factors and practices such as frequency and number of sexual partners, antibiotic use, personal hygiene, mode of underwear cleaning and the frequency of VVC infection. *C. albicans* isolates were more susceptible to amphotericin B and clotrimazole, although an appreciable number of the isolates showed some resistance. Studies on antifungal susceptibility of *C. albicans* should thus be done routinely before recommending antifungal therapy in vaginal candidiasis.

### Electronic supplementary material

Below is the link to the electronic supplementary material.

Supplementary Material 1

Supplementary Material 2

Supplementary Material 3

## Data Availability

Data from the research are available in the University Institutional Repository KNUST Space and the Faculty of Pharmacy and Pharmaceutical Sciences (Microbiology section), KNUST.
