# Supplementary material for: Resistant C. albicans implicated in recurrent vulvovaginal candidiasis (RVVC) among women in a tertiary healthcare facility in Kumasi, Ghana
Source: BMC Womens Health. 2024 Jul 19;24:412. doi: 10.1186/s12905-024-03217-6 (PMC11264716; doi:10.1186/s12905-024-03217-6)
Supplement: Supplementary file 1 — Supplementary Material 1 [file 12905_2024_3217_MOESM1_ESM.docx]

**Supplementary File 1 – Questionnaire used**

**DEMOGRAPHIC INFORMATION**

1. Age………..…
2. Where do you stay……………………
3. Educational Background: What is the highest grade you completed?

None [ ] Primary [ ] J.H.S. [ ] S.H.S. [ ] O LEVEL [ ] TERTIARY [ ]

1. Marital Status: Married [ ] Single [ ] Divorced [ ] Separated [ ] Consensual [ ]

**LEVEL OF IMMUNITY/IMMUNE SUPRESSION**

1. Do you suffer any chronic disease? Yes [ ] No [ ]
2. If yes what kind of chronic disease? Diabetes [ ] HIV AIDS [ ] Hypertension [ ] Others …………………………….
3. Are you pregnant? Yes [ ] No [ ]
4. How many times have you suffered vaginal infection in the last 12 months?

Never [ ] 1 time [ ] 1-3 times [ ] >3 times [ ] Others………………………………………

1. How many times have you suffered yeast infection in your life time?

Never [ ] <3 times [ ] 3-9 times [ ] 10-19 times [ ] 20-39 times [ ]

>40 times [ ] Others………………………………………………………

**TREATMENT PERCEPTION AND ATTITUDE**

1. What medication do you usually use when you have vaginal infection?

Amoxicillin [ ] Ciprofloxacin [ ] Clotrimazole [ ] Fluconazole [ ] Penicillin [ ] Herbal liquid preparation [ ] Specify ………………………. Herbal cream [ ] Specify………………………………

Others……………………………………………………………… Don’t know [ ]

1. For how long do you wait before you visit the hospital or Pharmacy for prescription when you have vaginal discharge signs and symptoms?

Less than 1 week [ ] 1 week [ ] 2 weeks [ ] 3-4 weeks [ ] Others ………………………

1. Where is your first point of call when you have vaginal discharge?

Pharmacy shop [ ] Nearest health center or hospital [ ] Herbal shop / center [ ]

Usage of left vaginal cream or drug [ ] Usage of left Herbal cream or drug [ ]

1. What drug is usually prescribed for you?

Amoxicillin [ ] Ciprofloxacin [ ] Fluconazole [ ] Clotrimazole [ ]

Herbal liquid preparation [ ] Specify …………………… Herbal cream [ ] Specify……………

Others………………………………………………………. Don’t know [ ]

1. Are you usually given antibiotics for treatment? Yes [ ] No [ ] don’t know [ ]
2. Do you know of antifungals? Yes [ ] Name one……………….. No [ ]

**INFECTION**

1. At what time do you realize you have vaginal thrush?

After sexual intercourse [ ] After visiting the toilet [ ] After wearing underwear [ ]

During the use of contraceptives [ ] After taking antibiotics or any kind of medication [ ]

Throughout pregnancy [ ] First trimester of pregnancy [ ] Second trimester of pregnancy [ ]

Third trimester of pregnancy [ ] Prior menstrual cycle [ ] During menstrual cycle [ ]

Post menstrual cycle [ ] Other…………………………………………………………………………

**PREDISPOSING FACTORS**

1. Does any of your family members suffer chronic or recurrent vaginal candidiasis (more than 3 times in a year)?

Yes [ ] No [ ]

1. Do you still experience signs and symptoms of candidiasis even after treatment? Yes [ ] No [ ]
2. Do you have any hormonal disorder? Yes [ ] No [ ]
3. Are you on any birth control method? Yes [ ] No [ ]

**TOILET FACILITY USAGE**

1. What kind of toilet facility do you frequently use?

Public water closet [ ] Public pit latrine [ ] Private water closet [ ] Private pit latrine [ ] Others

**PERSONAL HYGIENE**

1. When do you normally wash your underwear after use?

Immediately after removal [ ] A day after removal [ ] Two to three days after removal [ ]

A week or more after removal [ ] Others………………………………………………………….

1. Do you use detergents or disinfectants to wash your underwear?

Never [ ] Sometimes [ ] Always [ ] Others…………………………………………………………….

1. How do you usually dry your underwear’s? Indoors [ ] Outdoors not in the sun [ ] Outdoors in the sun [ ]
2. What underwear fabric do you use? Always cotton [ ] Mix of fabric [ ]Only synthetic [ ] Others……………
3. On a scale of 0-7 (low to high) on the average how often do you wear tight clothing, example jeans trousers?

0 [ ] 1 [ ] 2 [ ] 3 [ ] 4 [ ] 5 [ ] 6 [ ] 7 [ ]

1. Do you wipe or clean the vagina after urination? Never [ ] Sometimes [ ] Always [ ]
2. If ‘Yes’ to Q16, with what do you clean the vagina? Water [ ] Tissue [ ] Others …………..
3. How many times do you bath in a day? Once a day [ ] Twice a day [ ] Others………….
4. How do you wipe your anal area after emptying your bowels? Front to back [ ] Back to front [ ] Others ……
5. How often do you use feminine hygiene products? Never [ ]Sometimes [ ]Always [ ] Others………
6. What menstrual protection product do you use? Cloth [ ] Sanitary pad [ ] Others………

**DOUCHING PRACTICES**

1. What do you use in cleaning your vagina area during and after bathing? Only water[ ]Water and soap[ ] Antiseptics[ ] Feminine wash and vaginal creams [ ] Herbal preparation [ ] Others………………………………
2. Do you insert finger when washing the vagina? Never [ ]Sometimes [ ] Always [ ] Others……
3. Do you wash only the outside vagina area or inside the vagina. Inside [ ] Outside [ ] Both [ ] Others……

**SEXUAL PRACTICES**

1. Are you sexually active? Yes [ ] No [ ]
2. How many sexual partners do you have now? One [ ] Two or more [ ] None [ ]
3. How many sexual partners have you had in your life time? 1 [ ] 2-3 [ ] 4-6 [ ] >6 [ ] None [ ] Other………
4. Does your partner suffer penile discharge? Yes [ ] No [ ]
5. Does your partner experience itch and rash around the genital area? Yes [ ] No [ ]
6. What is your weekly frequency of sexual intercourse during the last 3 months?

Never [ ] <1 time a week [ ] 1-2 times a week [ ]3-6 times a week [ ] 7+ times a week [ ]Others...…

**USE OF ANTIBIOTICS**

1. How often do you use antibiotics? Never [ ]Sometimes (1/3) [ ]Frequently (2/3) [ ]Always [ ] Others……
2. How many times have you used antibiotics in the last 12 months?

1 time medication[ ]1-3 times medication [ ]3-5 times medication [ ] >5 times medication [ ] Others……

**PREDISPOSING FACTORS**

1. Does any of your family members suffer chronic or recurrent vaginal candidiasis (more than 3 times in a year)?

Yes [ ] No [ ]

1. Do you still experience signs and symptoms of candidiasis even after treatment? Yes [ ] No [ ]
2. Do you have any hormonal disorder? Yes [ ] No [ ]
3. Which birth control method are you on?

No method [ ] Oral contraceptive [ ] Diaphragm [ ] Spermicides [ ] Other methods [ ]

1. For how long have you been on contraceptive?

1-3 months [ ] 3-6 months [ ] 1-2year s [ ] >2years [ ] Not applicable [ ]
