# Supplementary material for: Resistant C. albicans implicated in recurrent vulvovaginal candidiasis (RVVC) among women in a tertiary healthcare facility in Kumasi, Ghana
Source: BMC Womens Health. 2024 Jul 19;24:412. doi: 10.1186/s12905-024-03217-6 (PMC11264716; doi:10.1186/s12905-024-03217-6)
Supplement: Supplementary file 2 — Supplementary Material 2 [file 12905_2024_3217_MOESM2_ESM.docx]

**Supplementary data**

Resistant *C. albicans* implicated in recurrent vulvovaginal candidiasis (rVVC) among women in a Tertiary Healthcare facility in Kumasi, Ghana.

Author details

Abena Kyeraa Sarpong^1^, Hayford Odoi^2^, Yaw Duah Boakye^1^, Vivian Etsiapa Boamah^1*^ Christian Agyare^1^

Author Affiliation

*^1Pharmaceutical Microbiology Section, Department of Pharmaceutics, Faculty of Pharmacy and Pharmaceutical Sciences, Kwame Nkrumah University of Science and Technology, Kumasi, Ghana^*

*^2Department of Pharmaceutical Microbiology, School of Pharmacy, University of Health and Allied Sciences, Ho, Volta Region, Ghana^*

Corresponding author

Boamah, VE. PhD.

Pharmaceutical Microbiology (FPPS)

KNUST

Kumasi-Ghana.

Tel: 00233244981167

Email: veboamah.pharm@knust.edu.gh; etsiapa@yahoo.com

**S1: Interpretive criteria of Zones of Inhibition for *C. albicans* as recommended by Clinical and Laboratory Standard Institute M44-A (CLSI M44-A)**

| **Antifungal agent** | **Disk content** | **Zone Diameter (mm)** | | |
| --- | --- | --- | --- | --- |
|  |  | **Sensitive (S)** | **Intermediate (I)** | **Resistance (R)** |
| Amphotericin B | 20µg | ≥ 15 | 14-11 | ≤ 10 |
| Fluconazole | 25 µg | ≥19 | 18-14 | ≤ 13 |
| Nystatin | 100 units | ≥ 15 | 14-11 | ≤ 10 |
| Miconazole | 10 µg | ≥ 20 | 19-11 | ≤ 10 |
| Clotrimazole | 10 µg | ≥ 15 | 14-11 | ≤ 10 |
| Voriconazole | 1 µg | ≥ 17 | 16-14 | ≤ 13 |
| 5’ Flucytosine | 1 µg | ≥ 15 | 14-11 | ≤ 10 |

Extracted from the CLSI website at clsi.org
