# Supplementary material for: Resistant C. albicans implicated in recurrent vulvovaginal candidiasis (RVVC) among women in a tertiary healthcare facility in Kumasi, Ghana
Source: BMC Womens Health. 2024 Jul 19;24:412. doi: 10.1186/s12905-024-03217-6 (PMC11264716; doi:10.1186/s12905-024-03217-6)
Supplement: Supplementary file 3 — Supplementary Material 3 [file 12905_2024_3217_MOESM3_ESM.docx]

Supplementary File 2 - Gel images


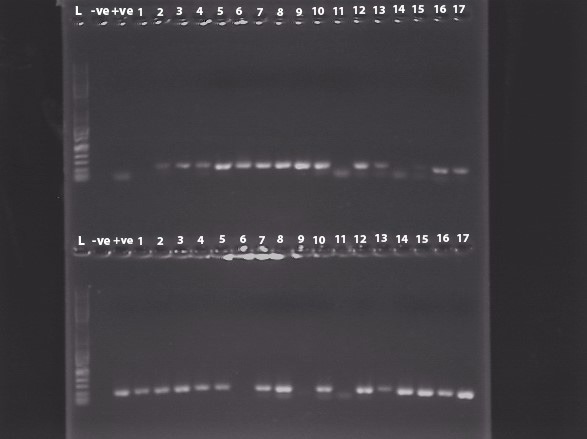


Amplified bands

175 bp

Amplified bands

Electrophoretic gel mage showing the 175bp PCR amplicon of the rRNA gene of *C. albicans*. L-100bp DNA ladder, -ve - Negative control, +ve - Positive control, 1 - 17 samples
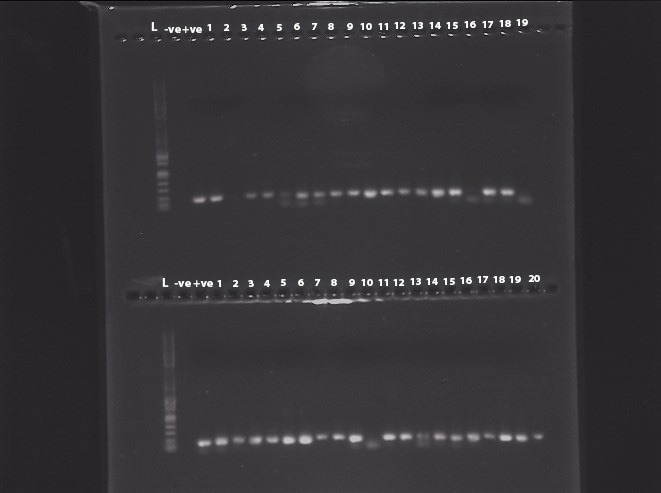


Amplified bands

Amplified bands

Electrophoretic gel mage showing the 175bp PCR amplicon of the rRNA gene of *C. albicans*. L-100bp DNA ladder, -ve - Negative control, +ve - Positive control, 1 - 20 samples
